# Multivariable analysis of bone mass reduction risk prediction and anxiety status in male HIV/AIDS patients

**DOI:** 10.3389/frph.2025.1703420

**Published:** 2026-01-16

**Authors:** Keke Hou, Tao Li, Shuyin Zhang, Jianglin He, Ting Wan, Yueqin Gao, Lei Xiong, Siqi Fu, Na Zhang, Guocheng Zhao

**Affiliations:** 1 Department of Radiology, Public Health Clinical Center of Chengdu, Chengdu, China; 2 Department of Radiology, The Fourth People’s Hospital of Chengdu, Chengdu, China; 3 The Clinical Hospital of Chengdu Brain Science Institute, MOE Key Lab for Neuroinformation, University of Electronic Science and Technology of China, Chengdu, China; 4 School of Life Science and Technology, University of Electronic Science and Technology of China, Chengdu, China

**Keywords:** HIV/AIDS, bone loss, risk prediction, anxiety, antiretroviral therapy (ART)

## Abstract

**Background:**

The psychological health issues and bone mass reduction observed in patients with human immunodeficiency virus (HIV)/acquired immunodeficiency syndrome (AIDS) have attracted increasing public attention. This study aimed to assess anxiety status in male HIV/AIDS patients, develop a risk prediction model for bone mass reduction, identify major contributing factors, and evaluate the predictive performance of the model.

**Methods:**

This cross-sectional study included 243 male HIV/AIDS inpatients who underwent dual-energy x-ray absorptiometry (DXA) at the Chengdu Public Health Clinical Medical Center, China, between March 2023 and December 2024. Clinical and laboratory data were retrospectively extracted from the hospital electronic medical record system, whereas anxiety information was prospectively assessed during hospitalization using the 14-item Hamilton Anxiety Rating Scale (HAM-A). Univariate and multivariate logistic regression analyses were performed to identify independent predictors of bone mass reduction, and a nomogram model was constructed and validated using the area under the curve (AUC), concordance index (C-index), Hosmer–Lemeshow test, and decision curve analysis (DCA).

**Results:**

1. HAM-A assessment of the 243 patients showed that 135 (55.56%) were likely to have anxiety, 73 (30.04%) were confirmed to have anxiety, 29 (11.93%) had marked anxiety, and 6 (2.47%) were likely to have severe anxiety. 2. Univariate analysis revealed that six variables—age, body mass index (BMI), antiretroviral therapy (ART), tenofovir disoproxil fumarate (TDF) exposure, hepatitis B surface antigen (HBsAg) positivity, and hepatitis C virus antibody (Anti-HCV) positivity—were significantly associated with bone mass reduction (*P* < 0.05). 3. Multivariate logistic regression analysis further confirmed these six variables as independent risk factors for bone mass reduction (*P* < 0.05). The AUC was 0.835 (95% CI: 0.775–0.894, *P* < 0.01), indicating good predictive performance. The bootstrap-validated C-index was 0.835, and the Hosmer–Lemeshow test (*P* = 0.483) demonstrated good calibration of the model. DCA showed that the model achieved favorable accuracy and net benefit across a wide range of threshold probabilities (0.04–0.90).

**Conclusion:**

Bone mass reduction in male HIV/AIDS patients is closely associated with multiple clinical factors, particularly the duration of ART and TDF exposure, age, BMI, and viral markers. In addition, the high prevalence of anxiety symptoms among these patients warrants clinical attention. The developed risk prediction model for bone mass reduction demonstrated good discrimination and calibration, providing an effective tool for clinical practice to identify high-risk patients and facilitate early intervention.

## Introduction

With the widespread clinical use of antiretroviral therapy (ART) and tenofovir disoproxil fumarate (TDF), treatment outcomes for patients with human immunodeficiency virus (HIV)/acquired immunodeficiency syndrome (AIDS) have improved significantly, and their survival has been markedly prolonged (1, 2). However, the psychological health needs of this population have become increasingly prominent (3–5). Anxiety disorder is the most common mental health problem among HIV/AIDS patients (5), increasing the risk of cardiovascular and cognitive complications and often accompanied by substance dependence. Therefore, early identification and timely intervention for anxiety are of great clinical importance.

HIV/AIDS also imposes a considerable burden on public health systems. Lifelong ART and comorbidity management require substantial medical resources. In China, as survival improves, the healthcare focus has shifted from infection control to chronic disease management. Thus, addressing complications such as anxiety and bone metabolism disorders is essential for improving overall health and optimizing resource use.

The use of ART and TDF (6) may adversely affect bone metabolism (7), increasing the risk of osteoporosis and fractures (8). Bone loss in HIV/AIDS involves complex mechanisms (9–12), including impaired osteoblast function (10) and chronic inflammation mediated by cytokines such as TNF-α, IL-6, and IFN-γ (11). The decline in bone mineral density is particularly evident in male patients (13). Therefore, identifying key risk factors for bone loss is crucial for protecting bone health. This study aimed to evaluate anxiety status, determine factors associated with bone mass reduction in male HIV/AIDS patients, and develop a multivariable prediction model to support early clinical intervention.

## Materials and methods

### Study population

This cross-sectional study was conducted at the Chengdu Public Health Clinical Medical Center, Chengdu, China, from March 2023 to December 2024. Clinical and laboratory data were retrospectively extracted from the electronic medical record (EMR) system, while anxiety information was prospectively collected during hospitalization. Patients were selected using a consecutive sampling method, meaning that all eligible male inpatients meeting the inclusion criteria during the study period were enrolled. The inclusion criteria were: (1) age over 18 years; (2) positive HIV test result; and (3) underwent DXA during admission.The exclusion criteria were: (1) presence of severe hepatic, renal, or thyroid disease; (2) diagnosis of malignant tumors; (3) long-term use of medications that may affect bone metabolism; and (4) incomplete clinical data.

Because the study aimed to include all eligible cases within the specified timeframe, no *a priori* sample size calculation was performed; the final sample (*n* = 243) represented all qualified patients during this period. A total of 243 eligible male HIV/AIDS patients were finally included in the study. The study was approved by the Ethics Committee of Chengdu Public Health Clinical Medical Center (Approval No.: YJ-K2025-06-01), and written informed consent was obtained from all participants.

### Research methods

#### Clinical data collection and anxiety assessment

Clinical data for all participants were retrieved from the EMR system, including HIV-related information and ART records. Height and weight were measured for each patient, and body mass index (BMI) was calculated by two trained researchers under the supervision of a clinical psychologist.

Psychological anxiety status was assessed face-to-face during hospitalization using the 14-item Hamilton Anxiety Rating Scale (HAM-A) (14), which covers both physical and psychological symptoms, such as tension, fear, insomnia, gastrointestinal, musculoskeletal, and respiratory symptoms. The Chinese version of the HAM-A, which has been validated for reliability and cultural adaptation, was used in this study without modification. Each symptom was rated from 0 (none) to 4 (severe). Based on the total score, anxiety status was classified as follows: 0–6 points, no anxiety; 7–13, possible anxiety; 14–21, definite anxiety; 22–28, marked anxiety; and ≥29, severe anxiety.

#### Bone mineral density (BMD) measurement

Bone mineral density for all patients was measured using a standardized DXA scanner (P11 PROD ADV FULL CN, GE Healthcare, Madison, WI). Scanning sites included the lumbar spine (L1–L4) and the femur (femoral neck and total hip).According to international standards (15), for men aged ≥50 years, T-scores were used: a T-score ≥ –1.0 was considered normal, and a T-score < –1.0 indicated bone mass reduction. For men aged <50 years, Z-scores were used: a Z-score ≤ –2.0 indicated bone mass reduction, and a Z-score > –2.0 was considered normal.Among the 243 male HIV/AIDS patients, 70 had normal bone mass, and 173 had bone mass reduction.

#### Viral load and CD4^+^ T-cell count

Plasma viral load was measured using the QUANTIPLEX™ b-DNA System 340 quantitative detection system (Bayer, USA), and peripheral blood CD4^+^ T-cell Count were determined using the EPICS-XL flow cytometer (Beckman Coulter, USA). Both viral load and CD4^+^ T-cell Count were obtained during the patients’ hospitalization.

#### Construction and evaluation of the risk prediction model

Univariate and multivariate logistic regression analyses were performed to identify independent predictors. Variables with statistical significance were then entered into a multivariate logistic regression model using the backward likelihood ratio method to construct the risk prediction model, and a nomogram was subsequently generated. The entry criterion was set at *P* < 0.05, and the removal criterion at *P* > 0.10.

The discriminative ability of the model was evaluated using the area under the receiver operating characteristic curve (AUC), while calibration was assessed with the Hosmer–Lemeshow goodness-of-fit test. The probability corresponding to the maximum Youden index was selected as the optimal threshold, and the decision curve analysis (DCA) was conducted to assess the clinical utility of the model.

### Statistical analysis

Statistical analyses were performed using SPSS version 28.0 (IBM Corp., USA) and R version 4.3.1 (https://www.r-project.org). The normality of continuous variables was assessed prior to analysis. Normally distributed variables were expressed as mean ± standard deviation and compared between groups using the independent samples t-test. Non-normally distributed variables were expressed as median and interquartile range (Q25, Q75) and compared using the Mann–Whitney *U* test. Categorical variables were expressed as frequency (percentage) and compared between groups using the chi-square test. For univariate analysis, the significance level was set at *α* = 0.10. Multivariate analysis was conducted using logistic regression with the backward likelihood ratio method. A *P* value < 0.05 was considered statistically significant.

## Results

### Baseline data

A total of 243 male HIV/AIDS patients were included in this study, with a mean age of 60.82 ± 12.73 years and a mean BMI of 23.74 ± 3.87 kg/m². The median time since HIV diagnosis was 36.00 (6.50, 84.00) months. Among all participants, 135 (55.56%) had tuberculosis and 73 (30.04%) were diagnosed with syphilis (Table 1). Regarding ART treatment, 212 patients had received antiretroviral therapy, and the incidence of bone mass reduction was higher in this group than in the 31 untreated patients (76.42% vs. 35.48%). Among the 156 patients whose treatment regimens included TDF, the incidence of bone mass reduction was higher compared with the 87 patients without TDF in their regimen (80.77% vs. 54.02%).

**Table 1:** Univariate analysis of bone mass loss in HIV/AIDS male patients.

Variables	Total (*n* = 243)	Normal bone mass Group(*n* = 70)	Bone loss group (*n* = 173)	t/Z/*χ*²	*p*-value
Age, mean ± SD (years)	60.82 ± 12.73	52.07 ± 15.08	64.36 ± 9.64	−7.570	<0.001
BMI, mean ± SD (kg/m^2^)	23.74 ± 3.87	22.34 ± 3.41	24.31 ± 3.91	−3.683	<0.001
Diagnosis time, median (Q25, Q75) (months)	36.00 (6.50, 84.00)	36.00 (2.00, 84.00)	39.00 (8.00, 84.00)	−1.148	0.251
viral load, median (Q25, Q75)(copies/mL)	83.30 (20.00, 61,050.00)	450.00 (20.00, 1,55,750.00)	73.90 (20.00, 29,000.00)	−1.159	0.247
CD4+ T count, median (Q25, Q75) (cells/mm^3^)	204.00 (84.50, 328.00)	203.50 (64.75, 333.50)	204.00 (90.00, 323.00)	−0.797	0.425
Accept ART, *N* (%)	212 (87.24)	50 (71.43)	162 (93.64)	22.094	<0.001
Accept TDF, *N* (%)	156 (64.20)	30 (42.86)	126 (72.83)	19.482	<0.001
HBsAg(+), *N* (%)	59 (24.28)	8 (11.43)	51 (29.48)	8.833	0.003
Anti-HCV (+), *N* (%)	39 (16.05)	6 (8.57)	33 (19.08)	4.081	0.043
Tumors, *N* (%)%	29 (11.93)	5 (7.14)	24 (13.87)	2.148	0.143
Kidney Failure, *N* (%)	49 (20.16)	12 (17.14)	37 (21.39)	0.558	0.455
Diabetes, *N* (%)	41 (16.87)	15 (21.43)	26 (15.03)	1.455	0.228
Hypertension, *N* (%)	24 (9.88)	7 (10.00)	17 (9.83)	0.002	0.967
Hypothyroidism, *N* (%)				−0.095	0.925
Tuberculosis, *N* (%)	135 (55.56)	38 (54.29)	97 (56.07)		
Syphilis, *N* (%)	73 (30.04)	24 (34.29)	49 (28.32)		
HAM-A scale	29 (11.93)	6 (8.57)	23 (13.29)		
Potentially anxiety, *N* (%)	6 (2.47)	2 (2.86)	4 (2.31)		

BMI, body mass index; ART, antiretroviral therapy; TDF, tenofovir disoproxil fumarate; HBsAg, Hepatitis B surface antig; Anti-HCV, anti-hepatitis C virus; HAM-A, Hamilton anxiety rating scale. All variables represent baseline characteristics of the total study population.

According to the HAM-A scale, 135 (55.56%) patients had mild anxiety symptoms (7–13 points), 73 (30.04%) had moderate anxiety (14–21 points), 29 (11.93%) had marked anxiety (22–28 points), and 6 (2.47%) had severe anxiety (≥29 points).

In Table 1, baseline clinical and laboratory characteristics are summarized, including HAM-A anxiety grading and BMD classification. All abbreviations are defined in the table footnote to ensure clarity.

### Construction of the bone mass reduction risk prediction model

Based on the univariate analysis results (Table 1), six variables—age, BMI, receipt of ART, receipt of TDF, HBsAg positivity, and Anti-HCV positivity—were entered into multivariate logistic regression. The results of multivariate logistic regression are presented in Table 2.

**Table 2:** Multivariate logistic regression analysis of bone loss in HIV/AIDS male patients.

Variables	B	SE	Wald	*P*	OR	95% CI
Age	0.078	0.015	28.29	<.001	1.081	1.05–1.112
BMI	0.105	0.053	3.967	0.046	1.111	1.002–1.232
Accept ART	1.420	0.595	5.702	0.017	4.138	1.29–13.276
Accept TDF	0.884	0.411	4.619	0.032	2.421	1.081–5.423
HBsAg(+)	0.943	0.462	4.163	0.041	2.567	1.038–6.349
Anti-HCV(+)	1.744	0.625	7.778	0.005	5.719	1.679–19.477
Constant	−8.302	1.589	27.313	<.001		

BMI, body mass index; ART, antiretroviral therapy; TDF, tenofovir disoproxil fumarate; HBsAg, hepatitis B surface antig; Anti-HCV, Anti-hepatitis C virus.

**Figure 1:**
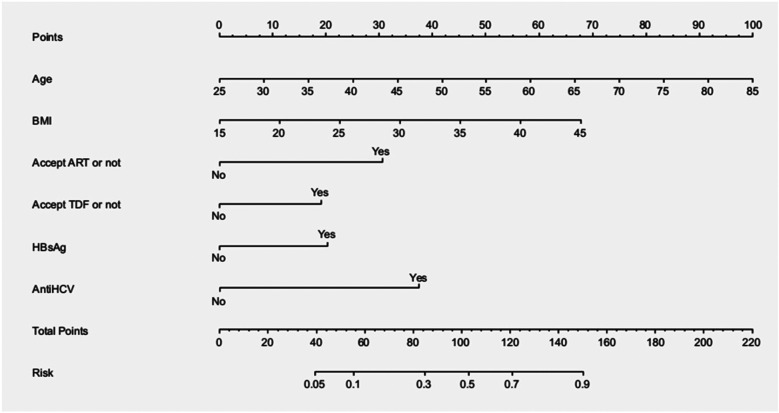
Column chart of bone loss nomogram-based risk prediction model for HIV/AIDS male patients.

The logistic regression results showed that increased age (OR = 1.081) and BMI (OR = 1.111), receipt of ART (OR = 4.318), receipt of TDF (OR = 2.421), HBsAg positivity (OR = 2.567), and Anti-HCV positivity (OR = 5.719) were independent risk factors for bone mass reduction (*P* < 0.05) (Table 2). On the basis of these variables, we constructed the following risk prediction model: X = −8.302 + 0.078 × age + 0.105 × BMI + 1.420 × ART + 0.884 × TDF + 0.943 × HBsAg(+) + 1.744 × Anti-HCV(+). Additionally, we created a nomogram based on this model (Figure 1) to help clinicians assess the risk of bone mass reduction for patients on the basis of their clinical indicators.

### Model evaluation and validation

**Figure 2:**
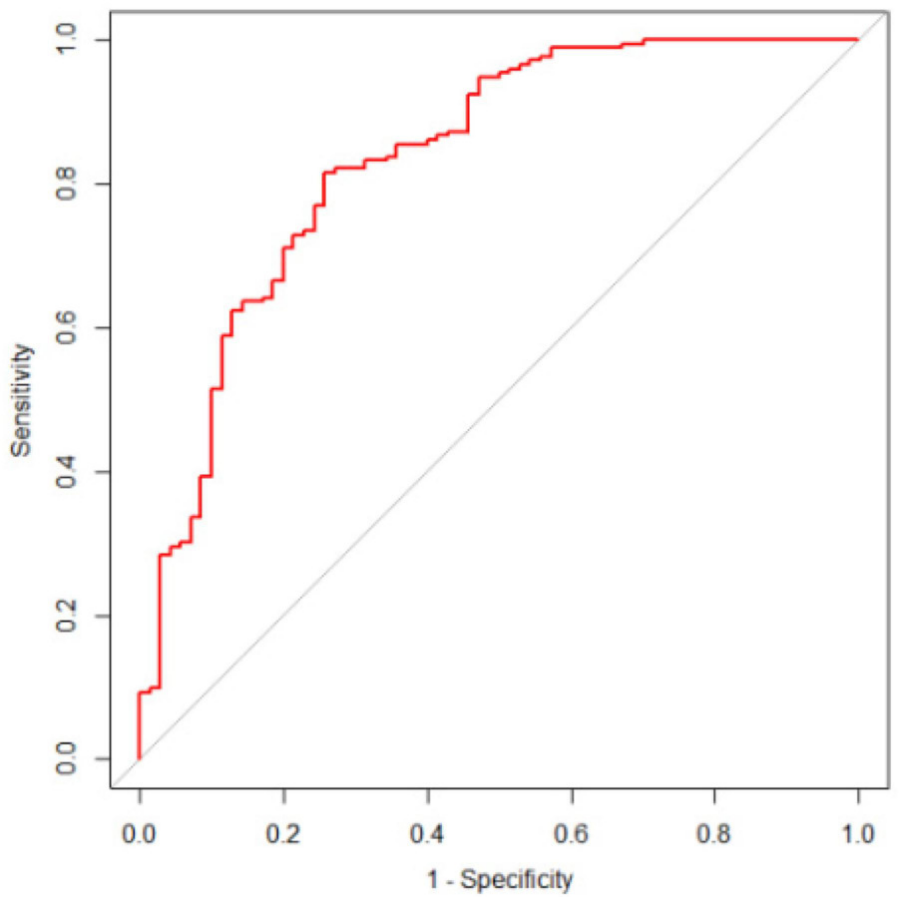
ROC curve of bone loss prediction model for HIV/AIDS male patients.

**Figure 3:**
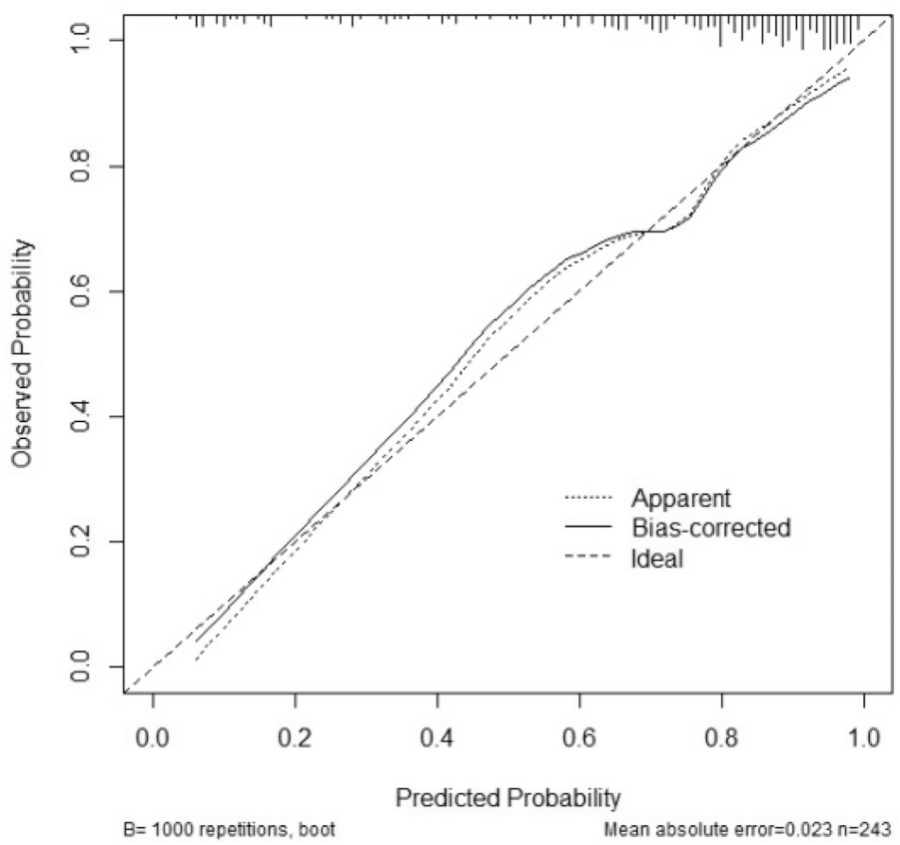
Calibration chart of bone loss prediction model for HIV/AIDS male patients.

**Figure 4:**
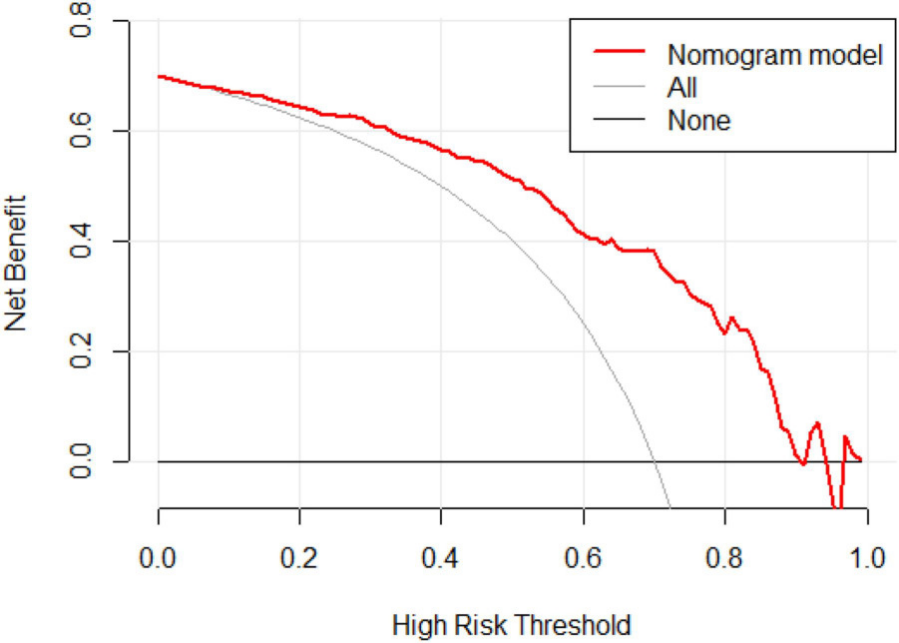
DCA curve of bone loss prediction model for HIV/AIDS male patients.

By calculating the predicted probabilities and using them as test variables, with the actual bone mass reduction status as the state variable, we evaluated the predictive performance of the model. ROC curve analysis revealed that the model's AUC was 0.835 (95% CI: 0.775–0.894, *P* < 0.01), indicating that the model had good discriminatory ability (Figure 2). The maximum Youden index was 0.558, with a sensitivity of 81.5% and specificity of 74.3%, and the threshold was set at 121.7. Furthermore, internal validation via the bootstrap method with 1,000 resamples demonstrated a C-index of 0.835 (95% CI: 0.771–0.886). The Hosmer‒Lemeshow test yielded a *χ*² value of 7.504, *P* = 0.483 (*P* > 0.05), indicating that the model had good calibration (Figure 3). Finally, DCA revealed that the model had high clinical accuracy and benefit when the threshold probability ranged from 0.04 to 0.90 (Figure 4).

## Discussion

The results of this study showed that factors such as age, BMI, ART, TDF, HBsAg positivity, and Anti-HCV positivity were closely associated with bone mass reduction, highlighting their importance in the management of HIV/AIDS patients. Based on these risk factors, we developed a risk prediction model for bone mass reduction in male HIV/AIDS patients, which may serve as a practical clinical risk assessment tool.

Multiple studies (6, 7, 14, 16) have reported a higher proportion of bone mass reduction among HIV patients receiving ART, which is consistent with the findings of this study. Although ART improves patients’ immune status, it may exert adverse effects on bone metabolism (17), leading to a decline in bone mineral density. The underlying mechanisms may involve disturbances in calcium–phosphorus metabolism, impaired osteoblast function, and increased osteoclast activity. Chronic immune activation, elevated levels of pro-inflammatory cytokines, and disordered vitamin D metabolism may also contribute to bone density loss. However, newer ART agents, such as dolutegravir, have shown minimal impact on bone metabolism, offering a potential new direction for optimizing treatment regimens (18).

There is a significant positive correlation between increasing age and declining bone mineral density (19, 20), as aging alters the metabolic processing of both exogenous and endogenous substrates in osteoblasts. Aging is associated with increased apoptosis and reduced numbers of osteoblasts and osteocytes, resulting in impaired mechanosensation, intercellular regulation, and exosome secretion, while enhanced bone resorption activity of macrophage-lineage cells further contributes to bone loss. These changes increase bone marrow fat content and oxidative stress, promoting osteoporosis. The mean patient age in this study was approximately 60 years, underscoring the importance of bone mass assessment as a key indicator of overall health status in male patients.

Fluctuations in BMI may influence bone mass and quality by altering the balance of bone metabolism. Previous studies have generally identified low BMI as an important risk factor for bone mass reduction and osteoporosis, as lower body weight often implies reduced fat mass and diminished mechanical support for bone; conversely, higher BMI may exert a protective effect (21, 22). However, other studies suggest that the impact of BMI on bone mineral density may vary with age, with high BMI in older populations potentially being associated with reduced bone quality (23). Consistent with these findings, our study indicates that elevated BMI in male HIV/AIDS patients may promote chronic inflammation, oxidative stress, and fatty infiltration of bone tissue through the action of pro-inflammatory adipokines and altered metabolism. These changes can impair bone formation and increase bone resorption, leading to reduced bone mineral density. Therefore, the influence of BMI on bone health in HIV patients may differ from that in the general population and warrants further investigation.

This study also found that HBV-positive patients often present with hepatic malnutrition, which can lead to increased osteoclast activity through pro-inflammatory cytokines that promote osteoclastogenesis, thereby resulting in bone loss. For example, interleukin-1 and tumor necrosis factor are associated with chronic inflammation, hepatic dysfunction, and liver fibrosis, all of which contribute to reduced lumbar spine and hip bone mineral density in patients with cirrhosis. HIV/HCV co-infected patients are at even greater risk of bone metabolic disorders, likely due to inflammatory cytokines such as TNF-α, which suppress bone formation while promoting bone resorption (8). In HIV-infected individuals, HCV infection may further exacerbate bone metabolism abnormalities by reducing bone formation and enhancing bone resorption. Moreover, the use of tenofovir disoproxil fumarate (TDF) in HBV-positive patients may contribute to bone mass reduction. Therefore, bone mass evaluation remains an essential clinical indicator for assessing overall health in HIV/AIDS patients co-infected with HCV and/or HBV.

HIV/AIDS patients are prone to experiencing anxiety (24), and anxiety itself may increase the risk of osteoporosis (25, 26). However, the findings of this study indicate that, among male HIV/AIDS patients, anxiety status was not independently and significantly associated with bone mass reduction. This may be because HIV-related factors such as antiretroviral therapy and chronic inflammation play a dominant role in bone metabolism, overshadowing the potential impact of anxiety. Similarly, Roebuck et al. reported that the association between anxiety and bone mineral density was not significant after excluding comorbid depression (26), suggesting that anxiety may not independently determine bone health in this population. Nevertheless, chronic anxiety may still indirectly influence health outcomes in HIV/AIDS patients by disrupting immune regulation through persistent activation of the hypothalamic–pituitary–adrenal axis, impairing CD4^+^ T-cell recovery, and increasing systemic inflammation. Anxiety can also reduce medication adherence and alter hormonal and metabolic pathways, thereby indirectly affecting bone metabolism. Continuous psychological assessment and timely intervention should therefore remain integral components of comprehensive management for people living with HIV.

Compared with traditional prediction models (27, 28), the model developed in this study integrates multiple clinical factors, including age, BMI, duration of ART and TDF treatment, and HBsAg and Anti-HCV status, enabling a more accurate risk assessment. Independent risk factors were identified through logistic regression analysis, ensuring the model's scientific rigor and reliability. Visualization through a nomogram simplifies clinical evaluation (29–31), helping clinicians quickly identify high-risk patients and design individualized interventions. The model showed good stability and practical utility across multiple validation methods, demonstrating its potential for clinical application.

This study has several limitations. First, only male HIV/AIDS patients were included, reflecting the current epidemiological profile in which men represent the majority of infected individuals. The limited number of female patients prevented gender-based comparisons, and women's higher susceptibility to anxiety may partly explain the absence of significant differences in anxiety status among male groups. Second, the mean age of participants was about 60 years, indicating underrepresentation of younger patients due to limited bone health awareness and screening among this group. Third, incomplete baseline data, missing family history, and lack of other psychosocial indicators may have introduced confounding bias. Finally, restricted access to DXA limited external validation of the model. Future studies should include patients of both sexes and wider age ranges, enlarge sample size, and strengthen multicenter collaboration to improve model stability and generalizability.

Despite these limitations, this study has notable strengths. It is among the few to integrate both psychological and metabolic parameters in modeling bone health among HIV/AIDS patients. The combination of prospectively collected anxiety data with objectively measured DXA parameters and validated clinical predictors enhances the scientific rigor and real-world applicability of the model. Furthermore, the model's good discrimination, calibration, and clinical utility support its potential use as a screening tool for early identification and intervention in high-risk male HIV/AIDS populations.

## Conclusion

The risk prediction model for bone mass reduction in male HIV/AIDS patients demonstrated good accuracy, calibration, and clinical utility, serving as a valuable tool for identifying high-risk individuals. Incorporating multiple variables—such as age, BMI, ART-related factors, and virological markers—the model highlights the importance of multidimensional risk assessment and supports individualized management strategies. Although anxiety was not an independent predictor, the high prevalence of psychological problems in this population warrants continued clinical attention. Future studies should further integrate mental health factors into bone metabolism research to promote comprehensive management and improve overall patient outcomes.

## Data Availability

The original contributions presented in the study are included in the article/Supplementary Material, further inquiries can be directed to the corresponding author.
